# CLOVER (CLOstridium difficile Vaccine Efficacy tRial) Study: A Phase 3, Randomized Trial Investigating the Efficacy and Safety of a Detoxified Toxin A/B Vaccine in Adults 50 Years and Older at Increased Risk of *Clostridioides difficile* Infection

**DOI:** 10.1093/cid/ciae410

**Published:** 2024-08-24

**Authors:** Curtis J Donskey, Erik R Dubberke, Nicola P Klein, Elizabeth G Liles, Katarzyna Szymkowiak, Mark H Wilcox, Jody Lawrence, Salim Bouguermouh, Haiying Zhang, Kenneth Koury, Ruth Bailey, Helen M Smith, Stephen Lockhart, Erik Lamberth, Warren V Kalina, Michael W Pride, Chris Webber, Annaliesa S Anderson, Kathrin U Jansen, William C Gruber, Nicholas Kitchin

**Affiliations:** Geriatric Research Education and Clinical Center, Louis Stokes Cleveland Veterans Affairs Medical Center, Cleveland, Ohio, USA; Division of Infectious Diseases, Washington University in St. Louis, St. Louis, Missouri, USA; Kaiser Permanente Vaccine Study Center, Oakland, California, USA; Kaiser Permanente Center for Health Research, Portland, Oregon, USA; Clinical Research, Synexus Polska Sp. z.o.o., Wroclaw, Poland; Leeds Teaching Hospitals NHS Trust and Leeds Institute of Medical Research, University of Leeds, Leeds, United Kingdom; Pfizer, Inc, Collegeville, Pennsylvania, USA; Pfizer, Inc, Pearl River, New York, USA; Pfizer, Inc, Collegeville, Pennsylvania, USA; Pfizer, Inc, Pearl River, New York, USA; Pfizer, Ltd, Hurley, United Kingdom; Pfizer, Ltd, Hurley, United Kingdom; Pfizer, Ltd, Hurley, United Kingdom; Pfizer, Inc, Collegeville, Pennsylvania, USA; Pfizer, Inc, Pearl River, New York, USA; Pfizer, Inc, Pearl River, New York, USA; Pfizer, Ltd, Hurley, United Kingdom; Pfizer, Inc, Pearl River, New York, USA; Pfizer, Inc, Pearl River, New York, USA; Pfizer, Inc, Pearl River, New York, USA; Pfizer, Ltd, Hurley, United Kingdom

**Keywords:** vaccine, *Clostridioides difficile*, infectious disease, adults, *Clostridioides difficile* infection

## Abstract

**Background:**

*Clostridioides difficile* infection (CDI) causes substantial mortality and healthcare burden. We assessed the detoxified toxin-A/B PF-06425090 vaccine for primary CDI prevention.

**Methods:**

This phase 3 observer-blinded study randomized (1:1) ≥50-year-olds at increased CDI risk (N = 17 535) to receive 3 PF-06425090 or placebo doses (0, 1, and 6 months). Primary end points were first CDI episode (≥3 unformed stools within 24 hours; central laboratory-confirmed toxin A/B positive) ≥14 days post-dose 3 (PD3; first primary) and post-dose 2 (PD2; second primary). CDI duration, need for CDI-related medical attention (secondary end points), and antibiotic use (post hoc analysis) PD3 were evaluated. Tolerability and safety were assessed.

**Results:**

The primary end point was not met (17 PF-06425090 and 25 placebo recipients had first CDI episode ≥14 days PD3 [vaccine efficacy (VE) = 31.0% (96.4% confidence interval [CI], −38.7% to 66.6%)]; 24 PF-06425090 and 34 placebo recipients had first CDI episode ≥14 days PD2 [VE = 28.6% (96.4% CI, −28.4% to 61.0%)]. Median CDI duration was lower with PF-06425090 (1 day) versus placebo (4 days; 2-sided nominal *P* = .02). Of participants with first CDI episode, 0 PF-06425090 and 11 placebo recipients sought CDI-related medical attention (post hoc analysis estimated VE = 100%; 95% CI, 59.6% to 100.0%) and 0 PF-06425090 and 10 placebo recipients required antibiotic treatment (VE = 100%; 95% CI, 54.8% to 100.0%). Local reactions were more frequent in PF-06425090 recipients, and systemic events were generally similar between groups; most were mild to moderate. Adverse event rates were similar between groups.

**Conclusions:**

Three PF-06425090 doses were safe and well tolerated. Although the primary end point was not met, PF-06425090 reduced symptom duration, CDI that required medical attention, and CDI-directed antibiotic treatment, highlighting its potential to reduce CDI-associated healthcare burden.

**Clinical Trials Registration:**

NCT03090191.

## Teaser

The phase 3 CLOVER study assessed the PF-06425090 vaccine for primary *Clostridioides difficile* infection (CDI) prevention in adults. Although the primary endpoint was not met, PF-06425090 reduced symptom duration, CDI requiring medical attention, and associated antibiotic treatment and was safe and well tolerated.

**(See the Editorial Commentary by Kuijper and Gerding on pages 1512–4.)**

*Clostridioides difficile* is a gram-positive, spore-forming bacillus [1]. *Clostridioides difficile* infection (CDI) can manifest from mild, self-limited diarrhea to life-threatening colitis [2–5]. After treatment, recurrent CDI occurs in 20%−30% of cases [6–8]. Recent antibiotic use, older age, and healthcare setting exposure are CDI-associated risk factors [1, 9–11].

Clinically significant CDI is associated with substantial mortality (eg, >20 000 US in-hospital deaths in 2017) and strains healthcare systems through increased healthcare costs, hospital length of stay, and resource utilization [7, 12–16]. In 2018, estimated excess mean CDI-associated US healthcare cost was $13 500/person within 2 months of diagnosis in ≥65-year-olds compared with matched controls; higher costs were linked to CDI-related hospitalization [15].

Although general downward trends in CDI incidence have occurred recently, only healthcare-associated CDI reductions were observed [16, 17]. Community-associated CDI incidence was stubbornly static, but now community-associated cases are common, surpassing US healthcare-associated cases in 2021 (55.9/100 000 versus 54.3/100 000) [16, 18]. CDI prevention efforts focus on people with healthcare facility admissions; no proven methods for preventing community-associated CDI exist, likely attributed in part to lack of clinical suspicion [19]. Strategies to further reduce CDI incidence are needed, particularly in cases that require medical intervention and community-associated CDI [19–21]. Accordingly, the US Centers for Disease Control and Prevention classified CDI as an urgent threat, outlining strategies for reducing CDI-associated healthcare burden, including antibiotic stewardship, improved diagnostics and therapeutics, and vaccine development [22].

Previous vaccine candidates failed to meet phase 3 primary objectives; although several candidates are in development, no preventative CDI vaccine is available [23–25]. PF-06425090 is a genetically detoxified toxin *C. difficile* vaccine formulated with modified (via amino acid substitutions) toxin A (TcdA) and B (TcdB) [24]. Because recombinant TcdA and TcdB demonstrated low residual toxicity levels, the proteins were subsequently chemically detoxified further to enhance vaccine safety [24]. In 2014, the US Food and Drug Administration granted PF-06425090 fast-track designation based on interim phase 2 data [24]. Phase 1 and 2 studies indicated that 3 PF-06425090 doses were safe and well tolerated and elicited robust TcdA and TcdB neutralizing antibody activity for ≤4 years post-vaccination in 50–85-year-olds [26–28].

Here, we report findings from the phase 3 randomized CLOVER (CLOstridium difficile Vaccine Efficacy tRial) trial, which assessed PF-06425090 efficacy for prevention of primary CDI in at-risk adults.

## METHODS

### Study Design and Participants

CLOVER was a global, phase 3, randomized, placebo-controlled study of efficacy and safety of a 3-dose PF-06425090 series for primary CDI prevention, which was conducted in 23 countries (382 sites) from March 2017 through December 2021 (the study sites are listed in the Supplementary Appendix). Participants were randomized 1:1 via interactive response to receive 0.5-mL aluminum hydroxide-containing PF-06425090 (200-µg total toxoid) or 0.5-mL saline (0.9% NaCl) placebo in the deltoid at 0, 1, and 6 months. In this observer-blinded study, staff who administered the vaccine were unblinded, but other site personnel and participants were blinded (detailed further in the Supplementary Appendix).

Eligible participants were ≥50-year-olds who were considered at increased CDI risk. Criteria for increased risk included nursing home or skilled nursing facility residency, having ≥1 inpatient hospitalization for ≥2 nights in the previous 12 months or scheduled ≥37 days after randomization, ≥2 emergency department visits or ≥10 outpatient visits in the previous 12 months, and systemic antibiotic receipt in the past 12 weeks. Participants with ≥1 confirmed prior CDI episode, prior *C. difficile* vaccination or monoclonal antibody therapy, known human immunodeficiency virus infection, any condition/treatment that resulted in frequent diarrhea, or the inability to respond to vaccination (eg, because of chronic systemic treatment with immunosuppressant medications or radiotherapy within 6 months) were excluded. The Supplementary Appendix outlines additional exclusion criteria and ethical study conduct.

### Sample Collection

Participants were given stool sample collection kits and shipping cooler boxes at their first visit, with instructions for collecting, packaging, and cooling stool samples. Whenever participants self-reported ≥3 unformed stools (Bristol stool chart types 5–7) in a 24-hour period, they were to collect a stool sample (third or subsequent unformed stool in 24-hour period) in their home or medical setting, regardless of whether presenting for diarrhea-related medical care. Participants were also to contact the study site, triggering ≥1 in-person visit and potential telephone follow-up. Frozen samples underwent central laboratory testing. Serum samples were obtained for immunogenicity testing during study visits before dose 1, 1 month post-dose 2, 1 month post-dose 3, and when potential CDI episodes occurred; presentation of these data is forthcoming.

### CDI Case Definition

Primary CDI episode was defined as having ≥3 unformed stools (Bristol stool chart types 5–7) within 24 hours and confirmed via a central laboratory as TcdB-positive via polymerase chain reaction (PCR) and free *C. difficile* toxin-positive via automated proprietary cell cytotoxic neutralization assay (CCNA) as described previously [29]. Recurrent CDI episode was defined as those that occurred ≤8 weeks after previous CDI episode onset, provided the previous episode symptoms resolved.

### Objectives and End Points

The primary efficacy objective was to demonstrate that PF-06425090 reduces first primary CDI episode incidence. The 2 primary end points were first CDI episode incidence ≥14 days post-dose 3 (first primary end point) and ≥14 days post-dose 2 (second primary end point).

Secondary objectives were evaluation of PF-06425090 efficacy in reducing incidence of all CDI episodes (primary and recurrent) and severity of first primary CDI episode. Secondary efficacy end points included incidence of all CDI episodes (primary and recurrent) post-dose 3, time to diarrhea resolution in the first primary CDI episode post-dose 3, and proportion of participants with first primary CDI episode that required CDI-related medical attention post-dose 3.

Vaccine efficacy (VE) for first primary CDI that required medical attention ≥14 days post-dose 3 and for first primary CDI ≥14 days post-dose 3 that required antibiotic use were evaluated as post hoc analyses.

The primary safety objective was to evaluate the PF-06425090 safety profile as measured by reactogenicity, adverse event (AE), and serious AE (SAE) rates. Local reactions and systemic events were reported by participants using electronic diaries ≤7 days post-dose (defined in Supplementary Table 1). AEs and SAEs were evaluated through 1 and 6 months post-dose 3, respectively. Unless otherwise stated, data to end of study are reported.

### Statistical Analyses

Sample size determination is outlined in the Supplementary Appendix. Per-protocol (PP) populations, which consisted of all randomized participants who received doses 1 and 2 (PP-2) or all 3 doses (PP-3) of PF-06425090 or placebo and had no major protocol violations ≤14 days after dose 2 (PP-2) or dose 3 (PP-3) administration, were used for efficacy evaluations.

A planned external data monitoring committee interim analysis was conducted after the first 30 primary CDI cases to determine whether the study should stop for efficacy or futility (Supplementary Appendix contains further details and results). For the final analysis, primary efficacy hypotheses were evaluated by testing the null hypothesis VE ≤20% using a fixed-sequence testing procedure, with a testing order of first primary CDI episode ≥14 days post-dose 3 and then ≥14 days post-dose 2. The null hypothesis would be rejected if the lower bound 96.4% confidence interval (CI) for VE was >20%. VE was estimated by 100 × (1 − IRR), where IRR is infection rate ratio of incidence rate in the PF-06425090 to placebo group. Type I error for final analyses was adjusted to α = 0.018 (1-sided) and α = 0.009 (1-sided) for primary and secondary efficacy end points, respectively, after accounting for multiplicity from interim and final analyses and because of multiple end points. Two-sided 96.4% and 98.2% CIs were presented for primary and secondary end points, respectively. Nominal 95% CIs were used for post hoc analyses. Clopper–Pearson-derived CIs for VE were adjusted for surveillance time. For the secondary end point of all CDI episodes post-dose 3 that involved identification of ≥1 case per recipient, VE and the corresponding CI were estimated with a proportional means model.

Safety and tolerability were evaluated in the safety population (all participants who received ≥1 PF-06425090 or placebo dose) using descriptive statistics. AE data were summarized according to first dose received.

## RESULTS

### Population

**Figure 1.**
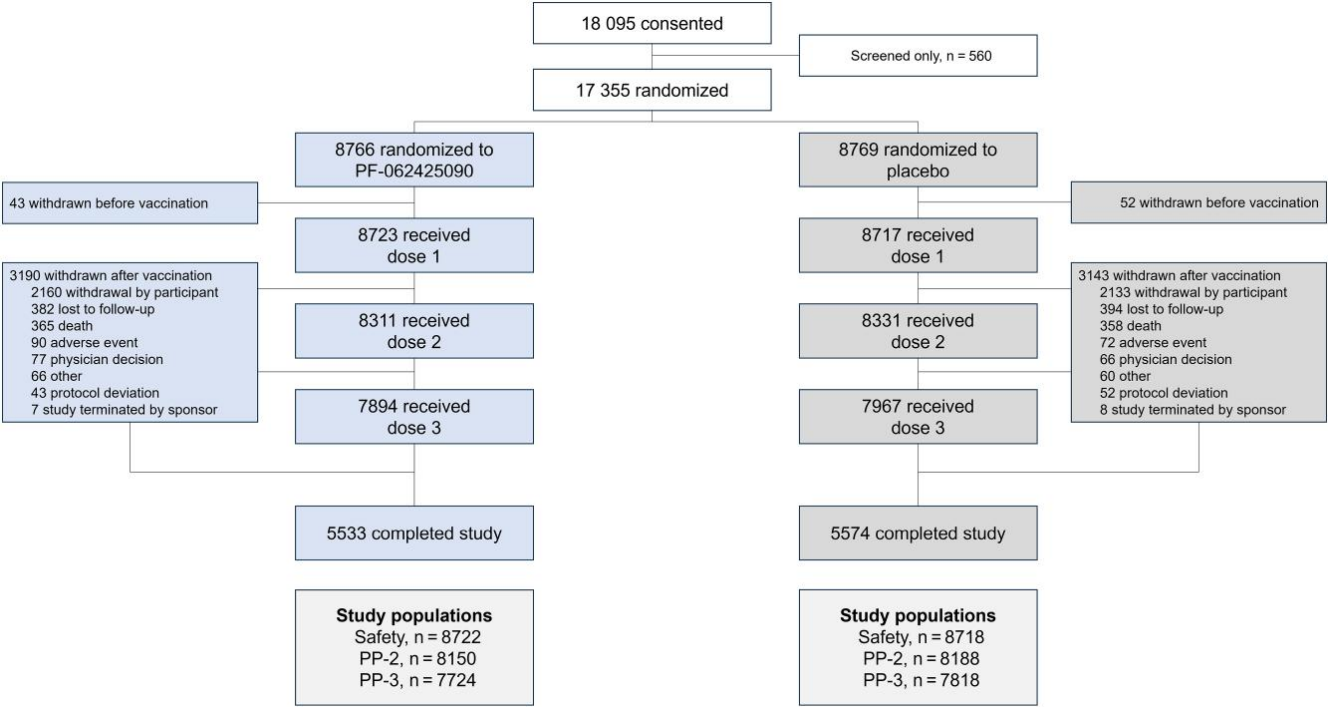
Participant disposition. Abbreviation: PP, per-protocol.

Overall, 17 535 participants were randomized to receive PF-06425090 (n = 8766) or placebo (n = 8769; Figure 1). Of those, 7894 (90.1%) in the PF-06425090 and 7967 (90.9%) in the placebo group received all 3 doses; 5533 (63.1%) and 5574 (63.6%) participants in the PF-06425090 and placebo groups completed the study, respectively. Mean follow-up duration post-dose 1 was similar in both study groups (PF-06425090, 37.5 months; placebo, 37.7 months). Demographic/baseline characteristics were generally similar between study groups (Table 1). Overall, 79.2% of participants were White, 51.5% female, and 65.1% from North America; mean age at randomization was 68 years. Most CDI cases were identified via home sample collection.

**Table 1.** Participant Demographics and Baseline Characteristics

Characteristic	PF-06425090 Vaccine (N = 8722)^a^	Placebo (N = 8718)^a^	Total (N = 17 440)
Sex, n (%)			
Female	4472 (51.3)	4501 (51.6)	8973 (51.5)
Male	4250 (48.7)	4217 (48.4)	8467 (48.5)
Race, n (%)			
White	6891 (79.0)	6922 (79.4)	13 813 (79.2)
Black	663 (7.6)	666 (7.6)	1329 (7.6)
American Indian or Alaska Native	53 (0.6)	54 (0.6)	107 (0.6)
Asian	754 (8.6)	725 (8.3)	1479 (8.5)
Native Hawaiian or other Pacific Islander	20 (0.2)	14 (0.2)	34 (0.2)
Other	327 (3.7)	322 (3.7)	649 (3.7)
Missing	14 (0.2)	15 (0.2)	29 (0.2)
Ethnicity, n (%)			
Hispanic/Latino	1119 (12.8)	1143 (13.1)	2262 (13.0)
Non-Hispanic/Non-Latino	7546 (86.5)	7515 (86.2)	15 061 (86.4)
Not reported	42 (0.5)	33 (0.4)	75 (0.4)
Unknown	15 (0.2)	27 (0.3)	42 (0.2)
Age at randomization, y			
Mean ± standard deviation	68.0 ± 7.5	68.1 ± 7.5	68.0 ± 7.5
Median (range)	68.0 (50.0–97.0)	68.0 (50.0–97.0)	68.0 (50.0–97.0)
Region, n (%)			
East Asia	664 (7.6)	662 (7.6)	1326 (7.6)
Europe	1915 (22.0)	1921 (22.0)	3836 (22.0)
North America	5684 (65.2)	5664 (65.0)	11 348 (65.1)
Oceania	126 (1.4)	132 (1.5)	258 (1.5)
South America	333 (3.8)	339 (3.9)	672 (3.9)

Participants demographics and baseline characteristics for the safety population.

^a^N was the number of participants who received each study intervention; N differed from the number of participants randomized to each study group (Figure 1) because 43 (PF-06425090) and 52 (placebo) participants withdrew before receiving any study vaccination, and 2 (PF-06425090) and 1 (placebo) participants were administered a different study intervention regimen after randomization than had been initially assigned.

### Efficacy

Of participants in the PF-06425090 and placebo groups, 8723 (99.5%) and 8717 (99.4%), respectively, received dose 1; 8311 (94.8%) and 8331 (95.0%) dose 2; and 7894 (90.1%) and 7967 (90.9%) all 3 doses. In the PP-2 population, 8150 (93.0%) and 8188 (93.4%) participants were included in the efficacy end point analyses in the PF-06425090 and placebo groups, respectively. There were 7724 (PF-06425090, 88.1%) and 7818 (placebo, 89.2%) participants included in the PP-3 population. Major protocol violations are described in the Supplementary Appendix.

**Figure 2.**
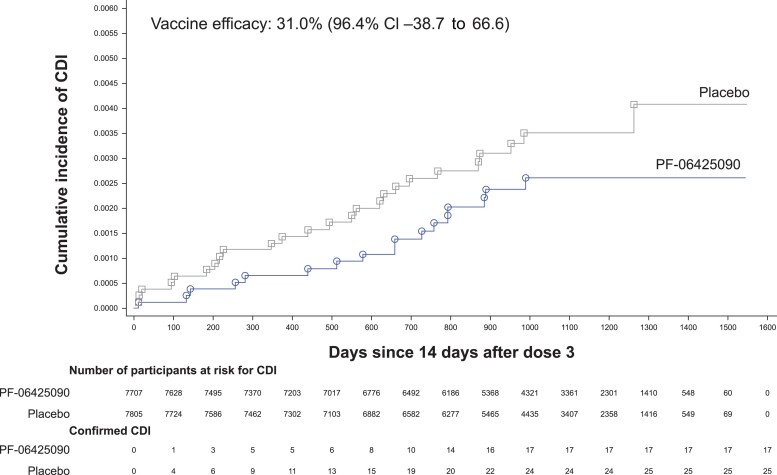
Cumulative incidence for the first primary episode of CDI ≥14 days after dose 3 (per-protocol [PP]-3 population). The PP-3 population included all randomized participants who received doses 1, 2, and 3 for the vaccine group to which they were randomized and had no major protocol violations up to and including 14 days after dose 3. The PP-2 population included all randomized participants who received doses 1 and 2 for the vaccine group to which they were randomized and had no major protocol violations up to and including 14 days after dose 2. Vaccine efficacy by cumulative time period is provided in Supplementary Table 2. Abbreviations: CDI, *Clostridioides difficile* infection; CI, confidence interval.

In the PP-3 population, 17 PF-06425090 and 25 placebo recipients, all of whom were from North America, had a first primary CDI episode ≥14 days post-dose 3, resulting in VE of 31.0% (96.4% CI, −38.7% to 66.6%; Table 2, Supplementary Table 2). Therefore, the primary efficacy end point criterion was not met. Separation of the cumulative incidence of CDI cases between the PF-06425090 and placebo groups was observed almost immediately ≥14 days post-dose 3 for the PP-3 population (Figure 2). In the PP-2 population, VE was 28.6% (96.4% CI, −28.4% to 61.0%), with 24 PF-06425090 and 34 placebo recipients having a first primary CDI episode ≥14 days post-dose 2.

**Table 2.** Vaccine Efficacy for the First Episode of *Clostridioides difficile* Infection Occurring ≥14 Days After Dose 3

Endpoint	PF-06425090 Vaccine (N = 7724)^a^		Placebo (N = 7818)^a^		Vaccine Efficacy (Confidence Interval), %
	n1^b^	Surveillance Time^c^ (n2^d^)	n1^b^	Surveillance Time^c^ (n2^d^)	
First episode of CDI^e^	17	21.18 (7707)	25	21.49 (7805)	31.0 (−38.7 to 66.6)
First episode of CDI that required medical attention^f,g^	0	21.24 (7720)	11	21.54 (7814)	100.0 (59.6 to 100.0)
First episode of CDI with antibiotic use^f^	0	21.24 (7720)	10	21.54 (7815)	100.0 (54.8 to 100.0)

Results are from the per-protocol 3 population; the per-protocol 3 population included all randomized participants who received doses 1, 2, and 3 of the study intervention to which they were randomized and had no major protocol violations up to and including 14 days after dose 3.

Abbreviation: CDI, *Clostridioides difficile* infection.

^a^Number of participants in the specified group.

^b^Number of CDI episodes that met the end point definition during the study.

^c^Total surveillance time in 1000 person-years for the given end point across all participants within each group. The period for episode accrual was ≥14 days after dose 3 to the end of the surveillance period.

^d^Number of participants at risk for the end point.

^e^Primary end point; the 2-sided 96.4% confidence interval (CI) was derived using the Clopper–Pearson method adjusted for surveillance time.

^f^Post hoc end point; the 2-sided nominal 95% CI was derived using the Clopper–Pearson method adjusted for surveillance time.

^g^Vaccine efficacy for this end point by cumulative time period is provided in Supplementary Table 2.

In the PP-3 population, 28 PF-06425090 and 32 placebo CDI episodes (primary or recurrent) occurred ≥14 days post-dose 3, providing an estimated VE of 11.1% (98.2% CI, −110.7% to 62.5%). Among the 17 PF-06425090 and 24 placebo recipients with first primary CDI episode ≥14 days post-dose 3, median CDI duration was 1 and 4 days (2-sided nominal *P* = .02), respectively. Five PF-06425090 and 3 placebo recipients had ≥1 recurrent CDI episode ≥14 days post-dose 3, providing an estimated VE of −69.3% (98.2% CI, −1533.1% to 75.6%).

Of the 0 PF-06425090 and 11 placebo recipients with first primary CDI episode ≥14 days post-dose 3 that required medical attention, post hoc assessment of estimated VE was 100% (95% CI, 59.6% to 100.0%; Table 2). In another post hoc analysis, 0 PF-06425090 and 10 placebo participants had first primary CDI ≥14 days post-dose 3 that required antibiotic intervention for CDI, yielding an estimated VE of 100% (95% CI, 54.8% to 100.0%; Table 2).

### Safety

**Figure 3.**
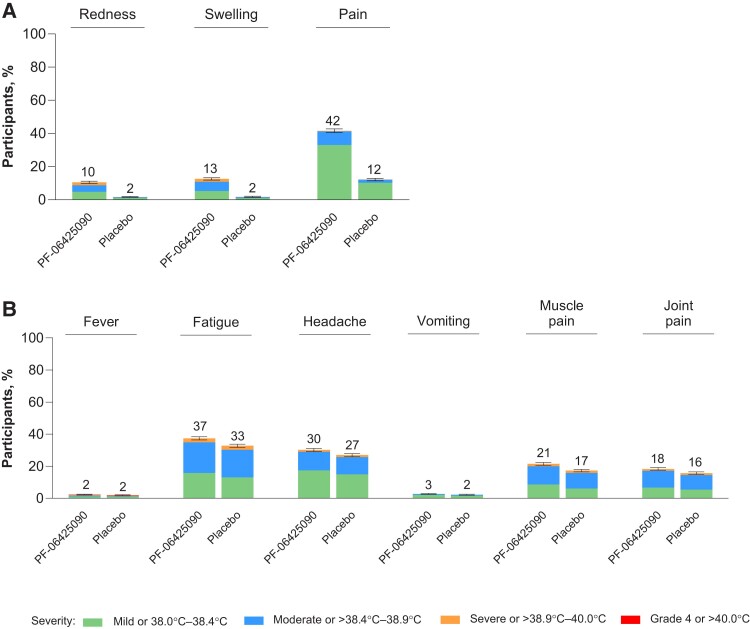
Local reactions (*A*) and systemic events (*B*) reported within 7 days after any vaccine dose. Results are from the safety population (PF-06425090, N = 8643; placebo, N = 8615). The severity scale is provided in Supplementary Table 1. The numbers above the bar are the percentage of participants with that local reaction or systemic event. Error bars are the 95% confidence intervals based on the Clopper–Pearson method. Severe local reactions after any dose were reported by 2.8% (244 of 8643) and 0.5% (42 of 8615) of participants in the PF-06425090 and placebo groups, respectively. One participant in the placebo group experienced a grade 4 local reaction (injection site pain). Severe systemic events after any dose were reported by 4.9% (423 of 8643) and 5.1% (437 of 8615) of participants in the PF-06425090 and placebo group, respectively. Grade 4 systemic events or fever >40.0°C were reported by 0.2% (17 of 8643) and 0.2% (18 of 8615) of participants in the PF-06425090 and placebo groups, respectively, and included fever >40.0°C (PF-06425090, 0.2% [15 of 8643]; placebo, 0.2% [18 of 8615]), fatigue (PF-06425090, <0.1% [1 of 8643]), muscle pain (PF-06425090, <0.1% [1 of 8643]), and joint pain (PF-06425090, <0.1% [1 of 8643]).

Percentages of participants in the PF-06425090 and placebo groups who reported reactogenicity events ≤7 days after each dose are shown in Figure 3. Most reactogenicity events were mild to moderate in severity. Injection-site pain and fatigue were the most common local reactions and systemic events, respectively, in both study groups. After any dose across study groups, ≤5.1% of participants reported severe reactogenicity events and ≤0.2% reported grade 4 events. For participants who received 3 PF-06425090 doses, median (range) onset of reactogenicity events was 2 days (1–7) and duration was 2 days (1–220) (Supplementary Table 3).

Percentages of participants reporting ≥1 AE were similar between study groups (PF-06425090, 52.1%; placebo, 51.0%; Table 3). Any related AE was reported by 3.5% (PF-06425090) and 1.7% (placebo) of participants. Differences in percentages of participants who reported any related AE refer to reactogenicity events also reported as AEs (eg, nausea, injection-site pain, redness, swelling, arthralgia, myalgia, headache).

**Table 3.** Summary of Adverse Events Reported to the End of the Study

Event	PF-06425090 Vaccine (N = 8722)^a^		Placebo (N = 8718)^a^	
	n^b^ (%)	No. of Events^c^	n^b^ (%)	No. of Events^c^
Any AE	4546 (52.1)	12 584	4443 (51.0)	12 058
Related	302 (3.5)	451	150 (1.7)	221
Severe or grade 4	1088 (12.5)	1889	1023 (11.7)	1745
SAEs	1297 (14.9)	2317	1267 (14.5)	2164
Related	7 (<0.1)	7	1 (<0.1)	3
Severe or grade 4	927 (10.6)	1506	873 (10.0)	1375
AEs that led to withdrawal	291 (3.3)	292	252 (2.9)	257
Related	8 (<0.1)	8	6 (<0.1)	8
Severe or grade 4	254 (2.9)	255	218 (2.5)	223
Death^d^	212 (2.4)	758	196 (2.2)	767

Results are from the safety population. Median (range) follow-up time after dose 3 was 35.6 months (0.0–51.8) and 35.7 months (0.3–51.9) in the PF-06425090 and placebo groups, respectively. Corresponding mean (standard deviation) follow-up times after dose 3 were 34.1 months (10.56) and 34.2 months (10.57).

Abbreviations: AE, adverse event; SAE, serious adverse event.

^a^N was the number of participants in the safety population who received each study intervention; N differed from the number of participants randomized to each study group (Figure 1) because 43 (PF-06425090) and 52 (placebo) participants withdrew before receiving any study intervention, and 2 (PF-06425090) and 1 (placebo) participants were administered a different study intervention regimen after randomization than what had been initially assigned.

^b^Number of participants who reported ≥1 of the specified events.

^c^Total number of events. Each participant may have reported multiple events. For death, n was the total number of AEs reported by participants who died.

^d^Death was considered an outcome, instead of an event. Number of events was an aggregate of all AEs experienced by participants who died.

SAEs were reported in 14.9% of PF-06425090 and 14.5% of placebo recipients (Table 3). Seven PF-06425090 recipients (<0.0009%) and 1 placebo recipient (<0.0002%) reported any SAEs considered study intervention related by the investigator. Related SAEs ≤6 months post-dose 3 occurred in the system organ classes of gastrointestinal disorders; general disorders and administration site conditions; musculoskeletal and connective tissue disorders; nervous system disorders; and respiratory, thoracic, and mediastinal disorders (Supplementary Table 4). Similar percentages of participants in both study groups experienced AEs that led to death (PF-06425090, 2.4% [n = 212]; placebo, 2.2% [n = 196]) and withdrew during the study for safety-related reasons (PF-06425090, 2.1% [n = 181]; placebo, 1.6% [n = 142]).

## DISCUSSION

CLOVER was a global, phase 3 efficacy study of PF-06425090 *C. difficile* vaccine in ≥50-year-olds at increased CDI risk. The primary objective was PF-06425090 efficacy in reducing incidence of first primary CDI episode. CDI end points involved active surveillance of stool samples that met study-defined diarrhea criteria; the participant and site were unaware of CDI testing results on these samples. The primary efficacy end point relied on stool samples undergoing central laboratory testing while the secondary end point of medically attended CDI involved participants seeking medical care as they would in real-world settings. A 2-step diagnostic approach was used, which included both PCR and CCNA assessments, consistent with international guidelines that recommend a 2-step diagnosis algorithm in which a sensitive screening assay (eg, PCR) is followed by a more specific assay for toxin [25] and captures the full CDI spectrum of case severities.

In CLOVER, estimated VE against first primary CDI episodes ≥14 days post-dose 3 was 31.0%, and the primary end point was not met because the success criterion of a 2-sided lower bound 96.4% CI was <20%. As CDI encompasses mild –to fulminant disease [2–5], this prospective study qualitatively captured a wide CDI spectrum, including mild self-limited disease. CDI cases otherwise missed were also captured as they are clinically insignificant, similar to seminal rotavirus vaccine findings that describe protective efficacy against clinically significant rotavirus diarrhea [30]. Soon after *C. difficile* was identified as the etiological agent of antibiotic-associated colitis, ≤20% of cases resolve spontaneously with offending antibiotic discontinuation [13, 31]. Although the primary end point was not met, secondary and post hoc analysis results indicated 3 PF-06425090 doses reduced median time to CDI resolution by 75%. Post hoc analyses also supported decreased need for medical attention (VE = 100%) and antibiotic use (VE = 100%) for first primary CDI episodes after 3 PF-06425090 doses. These post hoc analyses suggest clinically meaningful results, including potential prevention of community-onset cases that require medical intervention.

In CLOVER, most primary CDI cases identified in PF-06425090 recipients lasted for relatively short durations and, because none required medical attention, were likely mild. Clinically meaningful improvements in time to CDI resolution suggest PF-06425090 may alleviate CDI severity from moderate/severe to mild, self-limited disease. Furthermore, substantial reductions in need for CDI-associated medical attention and antibiotic use suggest participants who received PF-06425090 had mild CDI that resolved without requiring medical attention or antibiotic treatment or were asymptomatic carriers with alternative causes of diarrhea. Such mild, self-limiting cases not requiring medical attention would not normally be identified as CDI because diagnostic procedures would only be applied with medical consultation and, therefore, would not feature in public health data. Limited efficacy against cases not normally identified is therefore less clinically relevant; a case definition that includes seeking medical attention for diarrhea would be more appropriate in future VE studies. Together, these findings suggest that PF-06425090 may reduce overall disease burden by potentially reducing CDI severity in vaccine recipients and consequent need for medical interventions. Limiting the need for medical attention not only alleviates healthcare resource strains but reduces potential for antibiotic exposure, which may help mitigate increasing global threats of antimicrobial resistance [32, 33].

PF-06425090 had a favorable safety and tolerability profile, consistent with previous phase 1 and 2 studies [27, 28, 34]. Reactogenicity events were generally mild to moderate, and similar AE, SAE, related SAE, and withdrawal rates were observed between the PF-06425090 and placebo groups.

Study strengths include a population comprising only participants at increased CDI risk. Other strengths are the global design, which allowed for diversity in assessing efficacy and safety, and inclusion of participants from both healthcare and community settings. To our knowledge, this was the first prospective VE study to capture the entire CDI spectrum in a quantitative manner. Another strength was the 2-step diagnostic approach, which included both PCR and CCNA assessments.

Study limitations should be noted. CLOVER was not designed to distinguish between community- and healthcare-associated CDI. Stool samples were mostly collected at home by participants or their caregivers, potentially introducing imprecise assessment of stool quantity and characteristics. The primary efficacy end point relied on central laboratory stool sample testing, while the secondary end point of medically attended CDI involved participants seeking medical care as they would in real-world settings. These differences may have influenced study findings. Furthermore, CDI cases that did not require medical attention can be assumed to be mild and transient in nature and not reaching the stage of stool sampling in normal clinical practice as some cases are self-limited without requiring treatment. Additionally, some participants classified as having CDI may have been asymptomatic carriers with diarrhea from other causes. Although toxin detection by CCNA increases specificity of CDI testing [35], asymptomatic carriers of toxigenic *C. difficile* can also have positive toxin assay results [36, 37]. Additionally, because CCNA testing cannot be used to distinguish between TcdA- and TcdB-producing isolates, evaluation of the contribution of specific toxins to study outcomes was precluded. Other limitations include that the relationship between CDI and antibody levels and between CDI and isolates remains to be determined, and exhaustion of stool sample volumes prevented comprehensive analysis of the presence of other enteropathogens. Finally, although recurrent CDI frequency was similar in both study groups, limited case numbers precluded adequate study of recurrent CDI.

The study was conducted partly during the coronavirus disease 2019 pandemic, which affected certain study activities, including sample collection because of site closures, continued study participation, and in-person activities because of pandemic-related study staff restrictions. Because of confounders, including time since vaccination and small case numbers with wide CIs, a definitive assessment of the effect of the pandemic on study outcomes is limited. However, duration of follow-up was similar between study arms, suggesting minimal effect on study outcomes. Additionally, despite pandemic-related effects on study site activities, no substantial effect on study safety was noted, likely because protocol-defined safety assessments were completed before the pandemic.

In conclusion, CLOVER showed that a 3-dose PF-06425090 regimen was safe and well tolerated in ≥50-year-olds at increased CDI risk. Although the primary efficacy end point of reducing incidence of first primary CDI episode was not met, high efficacy was observed for PF-06425090 in preventing CDI cases that required medical attention and necessitated antibiotic treatment as well as CDI duration. Based on secondary end points and post hoc analyses, PF-06425090 showed potential to provide public health benefit by reducing CDI disease burden, including CDI cases severe enough to require medical attention and antibiotic treatment.

## Supplementary Data

Supplementary materials are available at *Clinical Infectious Diseases* online. Consisting of data provided by the authors to benefit the reader, the posted materials are not copyedited and are the sole responsibility of the authors, so questions or comments should be addressed to the corresponding author.

## Supplementary Material

ciae410_Supplementary_Data

## Notes

***Author Contributions.*** E. R. D., N. P. K., J. L., K. K., M. W. P., C. W., A. S. A., K. U. J., W. C. G., and N. K. contributed to the study concept and design. C. J. D., E. R. D., N. P. K., E. G. L., K. S., M. H. W., and J. L. contributed to data collection. E. R. D., J. L., H. Z., K. K., S. L., M. W. P., C. W., A. S. A., K. U. J., W. C. G., and N. K. contributed to the analysis and interpretation of the results. All authors contributed to draft development and reviewed and approved the final draft of the manuscript for submission.

***Acknowledgments.*** The authors thank the study participants and the CLOVER (CLOstridium difficile Vaccine Efficacy tRial) study team (see the Supplementary Appendix for the list of principal investigators). In addition, the authors thank the Kaiser Permanente Vaccine Center for their support in the trial's design and C. Hall Jones (Pfizer, Inc) for assay development. Medical writing support was provided by Tricia Newell, PhD, Kandyss Najjar, PhD, and Andrea Bothwell, BSc, of ICON (Blue Bell, PA) and was funded by Pfizer, Inc.

***Financial support*.** This work was supported by Pfizer, Inc

***Data sharing statement.*** Upon request and subject to review, Pfizer will provide the data that support the findings of this study. Subject to certain criteria, conditions and exceptions, Pfizer may also provide access to the related individual de-identified participant data. See https://www.pfizer.com/science/clinical-trials/trial-data-and-results for more information.

***Study protocol.*** The study protocol for this study is available at ClinicalTrials.gov at the following link: https://classic.clinicaltrials.gov/ct2/show/NCT03090191.
