# Supplementary material for: CLOVER (CLOstridium difficile Vaccine Efficacy tRial) Study: A Phase 3, Randomized Trial Investigating the Efficacy and Safety of a Detoxified Toxin A/B Vaccine in Adults 50 Years and Older at Increased Risk of Clostridioides difficile Infection
Source: Clin Infect Dis. 2024 Aug 24;79(6):1503–11. doi: 10.1093/cid/ciae410 (PMC11650871; doi:10.1093/cid/ciae410)
Supplement: ciae410_Supplementary_Data [file ciae410_supplementary_data.pdf]

APPENDIX

List of investigators*

Name	Institute	Location
Abbas, Jalal	Clinical Research Institute of Arizona	Surprise, AZ, USA
Acevedo, Armando	Acevedo Clinical Research Associates	Miami, FL, USA
Adams, Mark	Central Kentucky Research Associates	Lexington, KY, USA
Aguiar, Oricel	Next Phase Research Alliance	Miami, FL, USA
Ahlers, Kevin	Barrett Clinic	Omaha, NE, USA
Althoff, Amy (previously Epstein, Shara; previously Lee, Dong)	Drexel University College of Medicine Division of Infectious Disease Clinical Research Unit	Philadelphia, PA, USA
Andersson, Lars-Magnus	Sahlgrenska Universitetssjukhuset - Östra	Göteborg, Sweden
Andrews, Charles	Diagnostics Research Group	San Antonio, TX, USA
Aoun, Mickael	Institut Jules Bordet	Brussels, Belgium
Arif, Ahmed	AA MRC	Flint, MI, USA
Arpášová, Katarína	Dionea	Nové Zámky, Slovakia
Arya, Mark (Ktut)	Australian Clinical Research Network Maroubra Medical Centre	Maroubra, Australia
Athan, Eugene	Barwon Health	Geelong, Australia
Ayesu, Kwabena	Omega Research Consultants	Debary, FL, USA
Azorr, Michael	Columbia Research Group	Portland, OR, USA
Bakdash, Wa'el	Community Clinical Research Center	Anderson, IN, USA
Balabanska, Rozalina	Acibadem City Clinic Tokuda	Sofia, Bulgaria
Banerjee, Promila	Edward Hines Jr VA Hospital	Hines, IL, USA
Barcan, Laura	Hospital Italiano de Buenos Aires	Buenos Aires, Argentina
Barnes, Lawrence	Rame Group Practice	Torpoint, UK
Barnum, Otis	Barnum Medical Research	Natchitoches, LA, USA
Bartlett, Andrew	Christie Clinic	Champaign, IL, USA
Batarse, Rodolfo	Covigilant Research	Rancho Mirage, CA, USA
Baxter, David	Stockport NHS Foundation Trust	Stockport, UK
Belle-Isle, Jasmin	ALPHA Recherche Clinique	Québec, QC, Canada
Benjamin, Sabrina	Universal Research Group	Tacoma, WA, USA
Berhe, Mezgebe	North Texas Infectious Diseases Consultants	Dallas, TX, USA
Berova, Temenuzhka	''Medical Center-1-Sevlievo''	Sevlievo, Bulgaria
Blažek, Josef	Nemocnice Kyjov	Kyjov, Czech Republic
Block, Stanley	Kentucky Pediatric/Adult Research	Bardstown, KY, USA
Bochan, Markian	Infectious Disease of Indiana	Carmel, IN, USA
	St. Vincent Hospital & Health Care Center	Indianapolis, IN, USA
	St. Vincent Outpatient Treatment Center	Indianapolis, IN, USA
Boone, Gary	Paradigm Clinical Research Centers	La Mesa, CA, USA

Bouchard, Luc (previously Lachance, Pierre)	Centre de Recherche Saint-Louis	Québec, QC, Canada
Bradley, Paul	Meridian Clinical Research	Savannah, GA, USA
Bradley, Suzanne	VA Ann Arbor Healthcare System	Ann Arbor, MI, USA
Briskin, Toby	Velocity Clinical Research	Cleveland, OH, USA
Brockmyre, Andrew	Holston Medical Group	Bristol, TN, USA
Brook, Loren (previously Reynolds, Gorman; previously Yacyshyn, Bruce)	UC Health Physicians Office Clifton	Cincinnati, OH, USA
	University Of Cincinnati – Internal Medicine	Cincinnati, OH, USA
	University of Cincinnati Medical Center	Cincinnati, OH, USA
Browder, David	PMG Research of Rocky Mount	Rocky Mount, NC, USA
Buitrago, Martha	Snake River Research	Idaho Falls, ID, USA
Burgher, Abram	The Pain Center of Arizona	Peoria, AZ, USA
Butuk, David	Solaris Clinical Research	Meridian, ID, USA
Cadena Bonfanti, Andrés	Clínica de la Costa	Barranquilla, Colombia
Cadena-Zuluaga, Jose	South Texas Veterans Healthcare System	San Antonio, TX, USA
Campos Barker, María	Biomedica Research Group	Santiago, Chile
Cannon, Kevin	PMG Research of Wilmington	Wilmington, NC, USA
Cardona, Jose	Indago Research & Health Center	Hialeah, FL, USA
Carignan, Alex	CIUSSS de l'Estrie - CHUS	Sherbrooke, QC, Canada
Carvalho, Alexandre	Hospital de Braga	Braga, Portugal
Castañeda Sabogal, Alex Napoleon	Centro de Investigaciones Biomédicas - CIBIOM/Clinica Peruano Americana	Trujillo, Peru
Chalhoub, Fadi	Clinical Neuroscience Solutions	Jacksonville, FL, USA
Chang, Christopher	New Mexico VA Healthcare System	Albuquerque, NM, USA
Charvát, Jiří	Fakultní nemocnice v Motole	Praha, Czech Republic
Cheong, Hee Jin (previously Song, Joon Young)	Korea University Guro Hospital	Seoul, South Korea
Choi, Won Suk (previously Park, Dae Won)	Korea University Ansan Hospital	Ansan, South Korea
Christensen, Shane	J. Lewis Research / Foothill Family Clinic South	Salt Lake City, UT, USA
Christensen, Tom	Main Street Physician's Care – Waterway	Little River, SC, USA
Cíbik, Viliam	MUDr. Viliam Cíbik, PhD	Pruské, Slovakia
Clark, Rebecca	Layton Medical Centre	Blackpool, UK
Cobo Reinoso, Francisco Javier	Hospital Universitario Ramón y Cajal	Madrid, Spain
Cohen, Stuart	CTSC Clinical Research Center	Sacramento, CA, USA
	Lawrence J. Ellison Ambulatory Care Center	Sacramento, CA, USA
	One Community Health	Sacramento, CA, USA
	University of California, Davis Medical Center	Sacramento, CA, USA
Coleman, Stephen	J. Lewis Research / Foothill Family Clinic Draper	Draper, UT, USA
Cordasco, Edward (previously Baird, Ian)	OhioHealth Riverside Methodist Hospital<br>Remington-Davis	Columbus, OH, USA

Corrales-Medina, Vicente	The Ottawa Hospital Civic Campus	Ottawa, ON, Canada
Cotter, José	Hospital Senhora de Oliveira – Guimarães	Guimarães, Portugal
Cox, Jeremy (previously Marais, Henri)	Covigilant Research	Riverside, CA, USA
Cruz Saldarriaga, María Edelmira	Hospital Nacional Adolfo Guevara Velasco	Cusco, Peru
Cruz, Marilou	Premier Health Research Center	Downey, CA, USA
Curiac, Dan	Gothia Forum CTC/Sahlgrenska	Göteborg, Sweden
Dabrowska, Magda	Synexus Polska, Oddział w Warszawie	Warszawa, Poland
Davis, Matthew	Rochester Clinical Research	Rochester, NY, USA
de Hoon, Jan (previously Vandermeulen, Corinne)	UZ Leuven	Leuven, Belgium
de Looze, Ferdinandus	Austrials	Sherwood, Australia
de los Santos Gil, Ignacio	Hospital Universitario de la Princesa	Madrid, Spain
De Salvo, Maria	Centro Médico Dra. De Salvo	Buenos Aires, Argentina
De St. Maurice, Annabelle (previously Rubin, Zachary)	Ronald Reagan UCLA Medical Center	Los Angeles, CA, USA
	UCLA CTRC Outpatient Unit	Los Angeles, CA, USA
Dechkova-Novakova, Temenuzhka	Center for Mental Health – Ruse	Ruse, Bulgaria
Denham, Douglas	Clinical Trials of Texas	San Antonio, TX, USA
Désilets, Étienne	Centre Intégré de Santé et de Services Sociaux de la Montérégie - Center- Hôpital Charles-Le Moyne	Greenfield Park, QC, Canada
Dever, Michael	CNS Healthcare	Orlando, FL, USA
Dimitrov, Ivan (previously Krastev, Georgi)	"MHAT- Dr. Hristo Stambolski”	Kazanlak, Bulgaria
Dionne, Marc	Centre de recherche du CHU de Québec-Université Laval	Québec, QC, Canada
Dlouhý, Pavel	Krajská zdravotní	Ústí nad Labem, Czech Republic
Donskey, Curtis	Louis Stokes Cleveland VA Medical Center	Cleveland, OH, USA
Doust, Matthew	Hope Research Institute	Phoenix, AZ, USA
	The Pain Center of Arizona	Phoenix, AZ, USA
Drasnar, Tomáš	Nemocnice Slaný	Slaný, Czech Republic
Dubberke, Erik	Barnes-Jewish Hospital	St Louis, MO, USA
	Washington University School of Medicine	St Louis, MO, USA
Duffy, Brendan (previously Friedlander, Ira)	Aultman Cardiology Clinical Trials	Canton, OH, USA
Dybová, Lenka	Doktor Brno	Brno, Czech Republic
Eder, Frank	Meridian Clinical Research	Binghamton, NY, USA
Edwards, Christopher	North Alabama Research Center	Athens, AL, USA

(previously Hendrix, Ernest)		
Egelhof, Richard (previously Mehta, Praful)	Alliance for Multispecialty Research	Wichita, KS, USA
Eliasson, Ken	Avdelningen för kliniska prövningar, S-huset	Örebro, Sweden
El-Shahawy, Mohamed	Academic Medical Research Institute	Los Angeles, CA, USA
Ensz, David	Meridian Clinical Research	Sioux City, IA, USA
Eom, Joong Sik	Gachon University Gil Medical Center	Incheon, South Korea
Essink, Brandon	Meridian Clinical Research	Omaha, NE, USA
Falcone, Robert	Amici Clinical Research	Raritan, NJ, USA
Fatakia, Adil	Tandem Clinical Research	Marrero, LA, USA
Faulmann, Grit	Diabetologische Gemeinschaftspraxis Dr. Schaper & Dr. Faulmann	Dresden, Germany
Feuerstadt, Paul	Gastroenterology Center of Connecticut	Hamden, CT, USA
Finley, Jon (previously Segall, Nathan)	Clinical Research Atlanta	Stockbridge, GA, USA
Fitz-Patrick, David	East-West Medical Research Institute	Honolulu, HI, USA
Fling, John	University of North Texas Health Science Center at Fort Worth	Fort Worth, TX, USA
Fox, Bradley	Liberty Family Practice	Erie, PA, USA
Fragoso, Veronica	Texas Center for Drug Development	Houston, TX, USA
Frandsen, Brad	Sound Medical Research	Port Orchard, WA, USA
Freeman, Alan (previously Blumenau, Joe)	Synexus US	Dallas, TX, USA
Frei, Karen (previously Noureddine, Wassim)	Breakthrough Clinical Trials	San Bernardino, CA, USA
Freuler, Cristina	Hospital Alemán	Buenos Aires, Argentina
Fried, David	Velocity Clinical Research, Providence	East Greenwich, RI, USA
Friedenberg, Keith	Great Lakes Medical Research	Mentor, OH, USA
Fukue, Masataka (previously Takahashi, Toshiyuki)	Medical Corporation Tokushukai Koga General Hospital	Koga, Japan
Fukushima, Yasushi	Fukuwa Clinic	Chūō, Japan
Fussell, Suzanne	Long Beach Clinical Trials Services	Long Beach, CA, USA
Gagneux-Brunon, Amandine	Hôpital Nord-CHU Saint Étienne	Saint-Priest-en-Jarez, France
Gajdoš, Peter	Slezská nemocnice v Opavě	Opava, Czech Republic
Galler, Greg	Kelsey Research Foundation	Houston, TX, USA
Garcia-Diaz, Julia	Ochsner Center for Primary Care and Wellness	New Orleans, LA, USA
	Ochsner Clinic Foundation	New Orleans, LA, USA
Gareca, Marcelo	Lehigh Valley Health Network/Network Office of Research and Innovation	Allentown, PA, USA
Gebhardt, Bruce	Sterling Research Group	Cincinnati, OH, USA

(previously Mangu, Padma; previously Wenker, Matthew)		
Gentry, Andrew	Bozeman Health Clinical Research	Bozeman, MT, USA
	Bozeman Health GI Clinic	Bozeman, MT, USA
Gilfillan, Christopher (previously Simpson, Richard)	Eastern Health - Box Hill Hospital	Box Hill, Australia
Giot, Jean-Baptiste	CHU de Liège - Domaine universitaire du Sart Tilman	Liège, Belgium
Gonzalez Dorado, Alexander	Centro de Investigaciones - MedPlus Centro de Recuperación Integral	Bogotá, Colombia
Gonzalez Rojas, Yaneicy	Optimus U	Miami, FL, USA
Gonzalez, Misael	Doctors Research Institute	Miami, FL, USA
Górgolas Hernández-Mora, Miguel	Hospital Universitario Fundación Jiménez Díaz	Madrid, Spain
Greguš, Miloš	KM Management	Špitálska, Slovakia
Griffin, Paul	Mater Misericordiae Ltd & Mater Medical Research Institute	South Brisbane, Australia
Grigorova, Valentina	MC 'Cardiohelp'	Sofia, Bulgaria
Grimard, Doria	Intermed Groupe Santé	Chicoutimi, QC, Canada
Grubb, Stephen	Tabor City Family Medicine	Tabor City, NC, USA
Grunstra, Bernard	Internal Medicine and Pediatric Associates of Bristol	Bristol, TN, USA
	PMG Research of Bristol	Bristol, TN, USA
Guptill, Jeffrey	Duke Early Phase Clinical Research	Durham, NC, USA
Hall, Stephen	Emeritus Research	Camberwell, Australia
Hall, Timothy	Knowle House Surgery	Plymouth, UK
Hamada, Kensuke (previously Futakuchi, Sachiko and Tachi, Kosuke)	Kamiiida daiichi General Hospital	Nagoya, Japan
Hartman, Aaron	Virginia Research Center	Midlothian, VA, USA
Haselby, Ray (previously Hall, Matthew)	Marshfield Medical Center – Marshfield	Marshfield, WI, USA
Heller, Barry	Long Beach Clinical Trials	Long Beach, CA, USA
Helman, Laura	MOC Research	Mishawaka, IN, USA
Heuer, Marvin	HMD Research	Orlando, FL, USA
Holodniy, Mark	Veterans Affairs Palo Alto Health Care System	Palo Alto, CA, USA
Hong, Matthew (previously Harper, Wayne)	Wake Research Associates	Raleigh, NC, USA
Horcajada Gallego, Juan Pablo	Hospital del Mar	Barcelona, Spain
Howard, Timothy (previously McMurray, James)	Medical Affiliated Research Center	Huntsville, AL, USA
Hrebenár, Slavomír	Pľúcna ambulancia Hrebenár	Spišská Nová Ves, Slovakia
Hsueh, Kuang-Chieh	Kaohsiung Veterans General Hospital	Kaohsiung City, Taiwan
Hurley, Donald	Medical Research South	Charleston, SC, USA
Husain, Syed Nasir	Revival Research Institute	Sterling Heights, MI, USA

Igusa, Ryotaro	Ōsaki Citizen Hospital	Ōsaki, Japan
Ikegaya, Naoki (previously Kodaira, Makoto)	Yaizu City Hospital	Yaizu, Japan
Inoue, Ken	Seishinkai Inoue Hospital	Itoshima, Japan
Ivanov, Ivan	MC Medica Plus	Veliko Tarnovo, Bulgaria
Iwanaga, Kentaro	Saga-Ken Medical Centre Koseikan	Saga, Japan
Jackson, Christian	VA Loma Linda Healthcare System Clinical Research Group	Loma Linda, CA, USA
	VA Loma Linda Healthcare System Ambulatory Care System	Redlands, CA, USA
Jagarlamudi, Kiran	PMG Research of Salisbury	Salisbury, NC, USA
Jagielska-Plata, Małgorzata (previously Cesarz, Marek)	Synexus Polska, Oddział w Gdansku	Gdańsk, Poland
Jamal, Aamir	California Kidney Specialists	Covina, CA, USA
	California Kidney Specialists	San Dimas, CA, USA
	Usborne Family Medicine	Covina, CA, USA
Janoff, Edward	Clinical Trial Center University of Colorado	Aurora, CO, USA
Jeanfreau, Robert	MedPharmics	Metairie, LA, USA
Jenkins, Neil	Heart of England NHS Foundation Trust	Birmingham, UK
Johnson, David	Bay Pines VA Healthcare System	Bay Pines, FL, USA
Jorge, Rosa	Centro Hospitalar do Baixo Vouga	Aveiro, Portugal
Julien, Katie	J. Lewis Research – Jordan River Family Medicine	South Jordan, UT, USA
Jung, Dong Sik	Dong-A University Hospital	Busan, South Korea
Kageyama, Takashi (previously Akai, Akifumi)	Tokai Memorial Hospital	Kasugai, Japan
Karen, Igor	Ordinace všeobecného praktického lékaře	Benátky nad Jizerou, Czech Republic
Kashima, Masayuki	Japanese Red Cross Kumamoto Hospital	Kumamoto, Japan
Käßner, Frank	MECS Cottbus	Cottbus, Germany
Kaster, Steven	Wenatchee Valley Hospital & Clinics	Wenatchee, WA, USA
Keltai, Katalin	Semmelweis Egyetem II. Belgyógyászati Klinika	Budapest, Hungary
Kerschischnik, Saskia (previously Koenig, Hans-Joachim)	Emovis	Berlin, Germany
Kim, Hyo Youl	Yonsei University Wonju Severance Christian Hospital	Wonju-si, South Korea
Kim, Joshua (previously Regal, Paul and Vandeleur, James)	Paratus Clinical	Kanwal, Australia
Kim, Shin Woo	Kyungpook National University Hospital	Daegu, South Korea
Kim, Yang Soo (previously Woo, JunHee)	Asan Medical Center	Songpa-gu, South Korea
Klein, Nicola	Kaiser Permanente Daly City	Daly City, CA, USA
	Kaiser Permanente Roseville	Roseville, CA, USA
	Kaiser Permanente Sacramento	Sacramento, CA, USA

	Kaiser Permanente San Jose	San Jose, CA, USA
	Kaiser Permanente Santa Clara	Santa Clara, CA, USA
	Kaiser Permanente South Sacramento	Sacramento, CA, USA
	Kaiser Permanente Walnut Creek	Walnut Creek, CA, USA
Kobayashi, Hiroyuki (previously Yano, Harumi)	Mito Kyodo General Hospital	Mito, Japan
Kobayashi, Kazuki	Yokosuka Kyosai Hospital	Yokosuka, Japan
Koch, Mark	Texas Health Care	Fort Worth, TX, USA
(previously Watemberg, Isaac)	Ventavia Research Group	Fort Worth, TX, USA
Kokko, Satu	Oulu Vaccine Research Clinic	Oulu, Finland
Kolečkář, Pavel	Poličská nemocnice	Polička, Czech Republic
Koumarová, Šárka	Sarkamed	Slaný, Czech Republic
Kovashki, Krasimir	"SHATPD-Troyan"	Troyan, Bulgaria
Krátky, Adrián	Medak	Bratislava, Slovakia
Kubíková, Yveta	PULMO-MEDIK	Bojnice, Slovakia
Labuda, Joseph	Preferred Primary Care Physicians	Uniontown, PA, USA
Laguna Torres, Victor	Clínica Internacional	Lima, Peru
Launay, Odile (previously Loulergue, Pierre)	CIC Vaccinologie Cochin-Pasteur	Paris, France
Lee, Jacob	Hallym University Kangnam Sacred Heart Hospital	Seoul, South Korea
Lee, Jin Seo (previously Eom, Joong Sik)	Hallym University Kangdong Sacred Heart Hospital	Seoul, South Korea
Lee, Jin Soo	Inha University Hospital	Incheon, South Korea
Leen, Clifford	NHS Lothian, Western General Hospital	Edinburgh, UK
Lemaignen, Adrien (previously Bernard, Louis)	CHRU de Tours - Hôpital Bretonneau	Tours, France
Lenner, Egon	MEDILEX	Rimavská Sobota, Slovakia
Levitan, Diane	MultiCare Institute for Research & Innovation	Tacoma, WA, USA
Liao, Chun-Hsing	Far Eastern Memorial Hospital	New Taipei City, Taiwan
Liles, Elizabeth	Kaiser Permanente Center for Health Research	Portland, OR, USA
Lillestol, Michael	Lillestol Research	Fargo, ND, USA
Literáti-Nagy, Botond	DRC	Balatonfüred, Hungary
Looney, David	VA San Diego Healthcare System	San Diego, CA, USA
López-Contreras González, Joaquin	Hospital de la Santa Creu i Sant Pau	Barcelona, Spain
Lorch, Daniel	Florida Sleep Disorder Center of Brandon	Brandon, FL, USA
	Teradan Clinical Trials	Brandon, FL, USA
Lucasti, Christopher	South Jersey Infectious Disease	Somers Point, NJ, USA
Luttermann, Matthias	Studienzentrum Nordwest	Westerstede, Germany
Luu, Maxime	CHU Dijon Bourgogne - Hôpital François Mitterand	Dijon, France
Maeda, Itaru	Miyanomori Memorial Hospital	Sapporo, Japan
Manning, Rickey	PMG Research of Knoxville	Knoxville, TN, USA

Marques, Nuno Miguel	Hospital Garcia de Orta	Almada, Portugal
Márquez Velásquez, Juan	Instituto de Coloproctologia ICO	Medellín, Colombia
Marrett, Paula-Jane	Fowey River Practice	Fowey, UK
Martin, Earl	DM Clinical Research	Tomball, TX, USA
	Martin Diagnostic Clinic	Tomball, TX, USA
Martin, Thomas	Salem VAMC	Salem, VA, USA
Martínez Navarro, Encarna	EAP Centelles	Centelles, Spain
Matherne, Paul	MedPharmics	Gulfport, MS, USA
Matsumoto, Shuichi	Fukuoka Tokushukai Hospital	Kasuga, Japan
Merritt, Brock	Phoenix Clinical	Phoenix, AZ, USA
Metev, Hristo	SHATPPD Dr. Dimitar Gramatikov – Ruse	Ruse, Bulgaria
Meyers, Peter	ClinSite	Canton, MI, USA
Mikamo, Hiroshige (previously Yamagishi, Yuka)	Aichi Medical University Hospital	Nagakute, Japan
Mikušová, Tereza	Interna a kardiologie	Rožnov pod Radhoštěm, Czech Republic
Molina de Salazar, Dora	Asociación IPS Médicos Internistas de Caldas	Manizales, Colombia
Montero Alonso, Marta	Hospital Universitari i Politècnic La Fe	Valencia, Spain
Morová, Andrea (previously Mora, Marián)	SPAOLO	Stará Turá, Slovakia
Mota, Margarida	Centro Hospitalar Vila Nova de Gaia/Espinho	Vila Nova de Gaia, Portugal
Moya-Hechevarria, Jaynier	Pines Care Research Center	Pembroke Pines, FL, USA
Muñoz García, Patricia (previously Bouza Santiago, Emilio)	Hospital Universitario Gregorio Marañón	Madrid, Spain
Muñoz, Manuel	Hospital Dr. Hernan Henriquez Aravena	Temuco, Chile
Murray, Alexander	PharmQuest	Greensboro, NC, USA
Mussaji, Murtaza	LinQ Research	Pearland, TX, USA
Muthiah, Padmaja	VA Long Beach Healthcare System	Long Beach, CA, USA
Nadar, Venkatesh	Capital Area Research	Camp Hill, PA, USA
Nahass, Ronald	ID Care	Hillsborough, NJ, USA
Naito, Toshio	Juntendo University Hospital	Bunkyō, Japan
Nakagawa, Hidemitsu	Nozaki Tokushukai Hospital	Daitō, Japan
Nakamura, Fumiyasu	Omi Medical Center	Kusatsu, Japan
Nakatsuchi, Yukio	Social Medical Care Corporation Hose-kai Marunouchi Hospital	Matsumoto, Japan
Nannini, Esteban	Sanatorio Británico	Rosario, Argentina
Nawa, Toru	Fukuyama City Hospital	Fukuyama, Japan
Nguyen, Hien	VA Northern California Health Care System	Mather, CA, USA
Northland, Rebeca	Fundación Arriarán	Santiago, Chile
Nunez-Avila, Rafael	Precision Clinical Research	Sunrise, FL, USA

	Soma Medical Center	West Palm Beach, FL, USA
Nyirati, Gábor	Bajai Szent Rókus Kórház, II Belgyogyaszat	Baja, Hungary
Odio, Alberto	Alta California Medical Group	Simi Valley, CA, USA
Oleszak, Tomasz	Synexus Polska, Oddział w Poznaniu	Poznań, Poland
O'Marro, Steven	Springfield Clinic Infectious Diseases	Springfield, IL, USA
Oñate Gutiérrez, José	Centro Médico Imbanaco de Cali	Cali, Colombia
Orkwiszewska-Nalewajko, Anna	Synexus Polska, Oddzial w Gdyni	Gdynia, Poland
O'Sullivan, Matthew	Westmead Hospital	Westmead, Australia
Paavola, Pauliina	Kokkola Vaccine Research Clinic	Kokkola, Finland
Padareva, Marianna	MC ''Tsaritsa Yoanna''	Sliven, Bulgaria
Paluga, Stanislav	MEDIPA	Piešťany, Slovakia
Pap, Tibor	CRU Hungary	Encs, Hungary
Papp, Erzsébet	Somogy Vármegyei Kaposi Mór Oktató Kórház	Kaposvár, Hungary
Pardi, Darrell	Mayo Clinic	Rochester, MN, USA
Patel, Suchet	Meridian Clinical Research	Endwell, NY, USA
Patel, Vikas	University of Colorado Clinical & Translational Research Center Clinic	Aurora, CO, USA
	University of Colorado Clinical & Translational Research Center Outpatient Clinic	Aurora, CO, USA
	University of Colorado Clinical & Translational Research Center Outpatient Clinic	Aurora, CO, USA
Peck, Kyong Ran	Samsung Medical Center	Seoul, South Korea
Peterson, James	J. Lewis Research / Foothill Family Clinic	Salt Lake City, UT, USA
Pfeiffer, Christopher (previously Forrest, Graeme)	VA Portland Healthcare System	Portland, OR, USA
Phillips, Lawrence	Atlanta VA Medical Center	Decatur, GA, USA
Pickrell, Paul (previously Lucksinger, Gregg)	Tekton Research	Austin, TX, USA
Plimpton, Charles (previously Dedinsky, Brian; previously Gutierrez, Sylvia)	MedPharmics	Phoenix, AZ, USA
Poirier, Andre	Centre Integre Universitaire de Santé et de Services Sociaux de la Mauricie et du Centre du Québec	Trois-Rivières, QC, Canada
Polomski, Petar (previously Simova, Iana)	Acibadem City Clinic University Hospital	Sofia, Bulgaria
Polster, Scott	PMG Research of Hickory	Hickory, NC, USA
Poretz, Donald	Clinical Alliance for Research & Education – Infectious Diseases	Annandale, VA, USA
Price, David	Newcastle upon Tyne Hospitals NHS Foundation Trust	Newcastle upon Tyne, UK
Pullman, John	Mercury Street Medical Group	Butte, MT, USA
Purighalla, Raman	Preferred Primary Care Physicians	Pittsburgh, PA, USA
Raad, George	PMG Research of Charlotte	Charlotte, NC, USA

Raev, Dimitar	MHAT "Sveta Anna - Sofia"	Sofia, Bulgaria
Råhlen, Lennart (previously Moris, Linda)	Karolinska Trial Alliance, KTA Prim	Stockholm, Sweden
Ramón Torrell, Josep María	Hospital Universitari de Bellvitge	Barcelona, Spain
Randall, William	PriMed Clinical Research	Dayton, OH, USA
Rankin, Bruce	Accel Research Sites	DeLand, FL, USA
Reisinger, Emil	Universitätsmedizin Rostock, Abteilung für Tropenmedizin und Infektionskrankheiten	Rostock, Germany
Richmond, Peter	Linear Clinical Research	Nedlands, Australia
Rodriguez Ables, Lilia	Finlay Medical Research	Miami, FL, USA
Rombo, Lars	Infektionsmottagningen, Mälarsjukhuset	Eskilstuna, Sweden
Rosen, Jeffrey	AMR Miami	Coral Gables, FL, USA
Rosen, Robert	Progressive Medicine of the Triad	Winston-Salem, NC, USA
Rubino, John	PMG Research of Raleigh	Raleigh, NC, USA
Ruffin, Omari (previously Lee, Marcus)	Trinity Clinical Research	Tullahoma, TN, USA
Rupp, Richard	University of Texas Medical Branch	Galveston, TX, USA
Ryan, John	The Alverton Practise, St Clare Medical Centre	Penzance, UK
Saavedra Solis, Sonia	Centro de Investigacion Clínica del Sur Temuco	Temuco, Chile
Saiger, Salma	Research Across America	Mesquite, TX, USA
Salata, Robert	University Hospitals Cleveland Medical Center	Cleveland, OH, USA
Saleh, Jamshid	Paradigm Clinical Research Centers	Redding, CA, USA
Salvador Saguez, Francisco	Psicomed Esudios Médicos	Antofagasta, Chile
Sánchez Vallejo, Gregorio	Fundación Cardiomet CEQUIN	Armenia, Colombia
Sapundzhieva, Mirena	MHAT-Silistra	Silistra, Bulgaria
Schiefke, Ingolf	EUGASTRO / Praxis	Leipzig, Germany
Schroder, Derek	PMG Research of Cary	Cary, NC, USA
Schwarz, Tino	Klinikum Würzburg Mitte	Würzburg, Germany
Seas Ramos, Carlos	Hospital Cayetano Heredia	San Martín de Porres, Peru
Sekino, Hisakuni	Sekino Hospital	Toshima, Japan
Seppa, Ilkka	Turku Vaccine Research Clinic	Turku, Finland
Seppa, Ilkka (previously Sipila, Marjaana and Virta, Miia)	Tampere Vaccine Research Clinic	Tampere, Finland
Seppa, Ilkka (previously Forsten, Aino and Markkula, Jukka and Rossi, Maija)	Pori Vaccine Research Clinic	Pori, Finland
Shah, Neena	Parker Jewish Institute for Health Care & Rehabilitation	New Hyde Park, NY, USA
Shaikh, Obaid	VA Pittsburgh Healthcare System	Pittsburgh, PA, USA
Sheng, Wang-Huei	National Taiwan University Hospital	Taipei, Taiwan
Sheridan, Ray	Royal Devon & Exeter Hospital	Exeter, UK

Shimizu, Hideaki (previously Arakawa, Tomoharu)	Daido Clinic	Nagoya, Japan
Sigal, Helena	SIBAmed Studienzentrum	Leipzig, Germany
Sikorski, Piotr	Vitamed	Bydgoszcz, Poland
Silva Sieger, Federico Arturo	Fundación Cardiovascular de Colombia	Floridablanca, Colombia
Simeonov, Iliyan	MHAT "Sv. Nikolay Chudotvorets"	Lom, Bulgaria
Sipinska-Surzynska, Malgorzata	Synexus Polska, Oddział w Katowicach	Katowice, Poland
Smith, Kent	Advanced Clinical Research/Gut Whisperer	Riverton, UT, USA
Sohn, Jang-Wook	Korea University Anam Hospital	Seoul, South Korea
Sojo Dorado, Jesús	Hospital Universitario Virgen Macarena	Sevilla, Spain
Sokol, Roman	Privátna urologická ambulancia	Trenčín, Slovakia
Sousa Regueiro, María Dolores	Complejo Hospitalario Universitario de La Coruña	A Coruña, Spain
Spitale, Natalia (previously Minguez, Angel)	Hospital Nuestra Señora de la Misericordia	Córdoba, Argentina
Springub, Joachim	Praxis Dr. med. J. Springub / W. Schwarz Studienzentrum Nord-West	Westerstede, Germany
St-Amour, Eric	Q & T Research Outaouais	Gatineau, QC, Canada
Stašková, Mariá	STALERG	Košice, Slovakia
Stephens, Michael	Fleming Island Center for Clinical Research	Fleming Island, FL, USA
Stoychev, Kaloyan	UMHAT ”Dr. Georgi Stranski”	Pleven, Bulgaria
Straubing, Steve (previously Gupta, Anubha)	Southeast Clinical Research	Chiefland, FL, USA
Strauss, Guy	Multi-Specialty Research Associates	Lake City, FL, USA
Strehárová, Anna	Fakultná nemocnica Trnava	Trnava, Slovakia
Strout, Cynthia	Coastal Carolina Research Center	North Charleston, SC, USA
Studdard, Harry	Coastal Clinical Research	Mobile, AL, USA
Szymkowiak, Katarzyna (previously Jaźwińska-Tarnawska, Ewa)	Synexus Polska, Oddział we Wroclawiu	Wrocław, Poland
Täschner, Heidrun	Gemeinschaftspraxis Dr. med. Heidrun Taeschner und Dr. med. Susanne Bonigut	Leipzig, Germany
Terrelonge, Antonio	Ocean Blue Medical Research Center	Miami Springs, FL, USA
Tetiurka, Bogusław	Salmed	Łęczna, Poland
Thurman, Louise	IPS Research	Oklahoma City, OK, USA
Tiholov, Rumen	MHAT ''Sv.Ivan Rilski-Kozloduy''	Kozloduy, Bulgaria
Tobino, Kazunori	Iizuka Hospital	Iizuka, Japan
Toldy-Schedel, Emil	Budapesti Szent Ferenc Kórház	Budapest, Hungary
Toms, Graham (previously Symonds, Rehan)	Oak Tree Surgery	Liskeard, UK
Toro Rendón, Luis	Fundación Centro de Investigación Clínica - CIC	Medellín, Colombia
Trevino, Miguel	Innovative Research of West Florida	Clearwater, FL, USA

Tsvetkova-Nedkova, Galina	MHAT ''Sveta Petka"	Vidin, Bulgaria
Turner, Mark	Advanced Clinical Research	Meridian, ID, USA
Usdan, Lisa	Clinical Neuroscience Solutions	Memphis, TN, USA
Vale, Noah (previously Lasko, Benjamin and Toma, Azhar)	Manna Research	Toronto, ON, Canada
Varano, Susann	Clinical Research Consulting	Milford, CT, USA
Várkonyi, István	ClinTrial Audit	Debrecen, Hungary
Veazey, Lauren (previously Gutmann, James)	Deaconess Clinic Mt. Pleasant	Evansville, IN, USA
Velásquez Osorio, Juan	Hospital Pablo Tobón Uribe	Medellín, Colombia
Vélez Londoño, Juan	Fundación Valle del Lili	Cali, Colombia
Veli-jukka Anttila	Helsinki University Central Hospital	Helsinki, Finland
Vértes, András	Dél-pesti Centrumkórház - Országos Hematológiai és Infektológiai Intézet	Budapest, Hungary
Vilella i Morató, Anna	Hospital Clínic de Barcelona	Barcelona, Spain
Vladimirova-Kitova, Lyudmila	UMHAT “Sveti Georgi”	Plovdiv, Bulgaria
Vrbicky, Keith	Meridian Clinical Research	Norfolk, NE, USA
Wadsworth, Larkin	Sundance Clinical Research	St. Louis, MO, USA
Wakefield, Paul	PMG Research of Knoxville	Knoxville, TN, USA
Wang, Gary	Malcom Randall VA Medical Center	Gainesville, FL, USA
Watanabe, Hiroyuki	Fukui-Ken Saiseikai Hospital	Fukui, Japan
Welker, James	Luminis Health Anne Arundel Medical Center	Annapolis, MD, USA
White, Alexander	Progressive Medical Research	Port Orange, FL, USA
Wie, Seong-Heon	The Catholic University of Korea, St. Vincent's Hospital	Gyeonggi-do, South Korea
Wijewardane, Priyantha	Baptist Health Center for Clinical Research	Little Rock, AR, USA
	Baptist Health Rehabilitation Institute	Little Rock, AR, USA
Wilcox, Mark	St James University Hospital	Leeds, UK
Wilson, Jonathan	PMG Research of Winston-Salem	Winston-Salem, NC, USA
Wiselka, Martin	University Hospitals of Leicester NHS Trust	Leicester, UK
Witzke, Oliver	Universitätsklinikum Essen	Essen, Germany
Wofford, Scott	Arkansas Gastroenterology	North Little Rock, AR, USA
Wong, Titus	Vancouver Coastal Health Research	Vancouver, BC, Canada
Woods, Christopher	Durham VAMC	Durham, NC, USA
Yakish, Jack	Westminster Family Medicine	Erie, PA, USA
Yamaguchi, Yukihiro	Kenwakai Otemachi Hospital	Kitakyushu, Japan
Yanagawa, Tatsuo	Nerima General Hospital	Nerima, Japan
Yanez, Giralt (previously Paone, Douglas)	Charisma Medical and Research Center	Miami Lakes, FL, USA
Young, Michele (previously Nehaki, Negin)	Phoenix VA Health Care System	Phoenix, AZ, USA

Žáková, Dagmar	MUDr. Žáková	Trenčín, Slovakia
Závada, Filip	Oblastní nemocnice Příbram	Příbram I, Czech Republic

*Investigators who screened and randomized participants.

Additional exclusion criteria

Participants were excluded from the study if they were pregnant or breastfeeding. Further exclusion criteria included previous receipt of *C difficile* vaccination or monoclonal antibody therapy, prior bowel resection (excluding appendectomy), any bleeding disorder, anticoagulant therapy, receipt of blood products or immunoglobulins within 6 months before study enrollment, and participation in other investigational drug or vaccine research studies within 28 days before study enrollment and until 1 month after dose 3. Individuals with any contraindication to vaccination or the *C difficile* vaccine components and those with other acute or chronic conditions that may have increased the risk of study participation or interfered with interpretation of results were also excluded.

Study blinding

The study staff dispensing and administering the vaccine were unblinded, but all other site study personnel, including the principal investigator and the participant, were blinded. Those study team members involved in ensuring that protocol requirements for investigational product handling, allocation, and administration were fulfilled at the site were unblinded for the duration of the study. All other study team members and all laboratory personnel performing the stool and serology assays remained blinded to vaccine assigned or received throughout the study.

Ethical study conduct

The study was conducted in accordance with ethical principles in the Declaration of Helsinki and applicable International Conference on Harmonisation Good Clinical Practice Guidelines, and all local regulatory requirements. All participants provided written informed consent.

Sample size determination

This study was placebo-controlled, event-driven, and randomized with a 1:1 randomization ratio between participants receiving PF-06425090 and placebo. With assumptions of a true adjusted vaccine efficacy (VE) of 66.5% after 3 doses of investigational product, 66 first primary CDI cases would provide 89.8% power to conclude true VE of >20%, taking into consideration 3 interim analyses at 30, 40, and 50 cases during the study. The early efficacy stopping boundaries were based on a group-sequential design utilizing a single-binomial distribution and gamma[-2] alpha spending function.

It was anticipated that up to approximately 17,476 adults, 50 years and older, might be enrolled globally to accumulate 66 first primary CDI cases. As this was an event-driven and group-sequential study, the total enrollment number might vary depending on the assumptions of incidence rate of the primary endpoint, true VE, and potential early stopping for efficacy or futility.

Interim analysis

A planned interim analysis was conducted after accruing 30 cases of first primary CDI in the PP-3 population in December 2020 and presented to an external data monitoring committee (EDMC) to determine whether the study should continue.

The sponsor was blinded to the results obtained at the interim analysis and was informed by the EDMC only that their recommendation was to continue the study. After the study was stopped and unblinded, however, results obtained at this interim analysis became available: VE in the PP-3 population at the time of the interim analysis was approximately 49.4% (98.8% CI: −38.4%, 83.5%) with 10 PF-062425090 and 20 placebo recipients having a first primary CDI episode. This led to the EDMC recommendation to continue the study. However, because of slow accrual of CDI cases and increased participant withdrawals over time, the study was stopped when ≥40 first primary CDI cases in the PP-3 population had accrued.

Major protocol violations

Major protocol violations ≤14 days post-dose 2 occurred in 285 (PF-06425090, n=148; placebo, n=137) and post-dose 3 occurred in 311 (n=166; n=145) participants; these participants were respectively excluded from PP-2 and PP-3 populations.

Table S1. Severity grading scale for local reactions and systemic events

	Mild	Moderate	Severe	Potentially life threatening (grade 4)
**Local reactions**				
Pain	Does not interfere with activity	Interferes with activity	Prevents daily activity	ED visit or hospitalization
Erythema/redness	2.5–5.0 cm (5–10 measuring device units)	>5.0–10.0 cm (11–20 measuring device units)	>10 cm (≥21 measuring device units)	Necrosis or exfoliative dermatitis
Induration/swelling	2.5–5.0 cm (5–10 measuring device units)	>5.0–10.0 cm (11–20 measuring device units)	>10 cm (≥21 measuring device units)	Necrosis
**Systemic events**				
Vomiting	1–2 times in 24 hours	>2 times in 24 hours	Requires IV hydration	ED visit or hospitalization
Headache	No interference with activity	Some interference with activity	Significant; prevents daily activity	ED visit or hospitalization
Fatigue/tenderness	No interference with activity	Some interference with activity	Significant; prevents daily activity	ED visit or hospitalization
Muscle pain	No interference with activity	Some interference with activity	Significant; prevents daily activity	ED visit or hospitalization
Joint pain	No interference with activity	Some interference with activity	Significant; prevents daily activity	ED visit or hospitalization

ED=emergency department; IV=intravenous.

Table S2. Vaccine efficacy by cumulative time period (PP-3 population)

Period	PF-06425090 vaccine (N[a]=7724)		Placebo (N[a]=7818)		VE (95% CI[e])
	n1[b]	Surveillance time[c] (n2[d])	n1[b]	Surveillance time[c] (n2[d])	
**First primary episode of CDI ≥14 days after dose 3**					
≤12 months	5	7.41 (7707)	10	7.50 (7805)	49.4% (–62.6, 86.4)
≤24 months	10	14.21 (7707)	19	14.40 (7805)	46.7% (–20.6, 77.8)
≤36 months	17	19.37 (7707)	24	19.65 (7805)	28.1% (–39.5, 63.8)
All	17	21.18 (7707)	25	21.49 (7805)	31.0% (–33.0, 65.0)
**First primary episode of CDI requiring medical attention ≥14 days after dose 3**					
≤12 months	0	7.42 (7720)	4	7.51 (7814)	100.0% (–53.3, 100.0)
≤24 months	0	14.24 (7720)	7	14.42 (7814)	100.0% (29.7, 100.0)
≤36 months	0	19.42 (7720)	10	19.70 (7814)	100.0% (54.8, 100.0)
All	0	21.24 (7720)	11	21.54 (7814)	100.0% (59.6, 100.0)

The PP-3 population included all randomized participants who received doses 1, 2, and 3 of the vaccine group to which they were randomized and had no major protocol violations up to and including 14 days after dose 3. CDI, *Clostridioides difficile* infection; PP, per protocol; VE, vaccine efficacy.

[a]Number of participants in the specified group.

[b]Number of episodes meeting the endpoint definition during the study.

[c]Total surveillance time in 1000 person-years for the given endpoint across all participants within each group. The time period for episode accrual was ≥14 days after dose 3 to the end of the surveillance period.

[d]Number of participants at risk for the endpoint.

[e]The 2-sided CI was derived using the Clopper-Pearson method adjusted for surveillance time.

Table S3. Median onset and duration of local reactions and systemic events (safety population)

	PF-06425090 vaccine		Placebo	
	**Median (range) onset, days**	**Median (range) duration, days**	**Median (range) onset, days**	**Median (range) duration, days**
Local reactions				
Redness				
Dose 1	2 (1–7)	1 (1–40)	1 (1–7)	1 (1–27)
Dose 2	2 (1–7)	2 (1–65)	2 (1–7)	1 (1–19)
Dose 3	2 (1–7)	2 (1–28)	2 (1–7)	1 (1–14)
Swelling				
Dose 1	2 (1–7)	1 (1–39)	1 (1–7)	1 (1–27)
Dose 2	2 (1–7)	2 (1–78)	1 (1–5)	1 (1–10)
Dose 3	2 (1–7)	2 (1–64)	1 (1–6)	1 (1–13)
Injection site pain				
Dose 1	2 (1–7)	1 (1–91)	1 (1–7)	1 (1–37)
Dose 2	2 (1–7)	2 (1–34)	1 (1–7)	1 (1–35)
Dose 3	2 (1–7)	2 (1–220)	1 (1–7)	1 (1–67)
Any				
Dose 1	2 (1–7)	1 (1–91)	1 (1–7)	1 (1–37)
Dose 2	2 (1–7)	2 (1–78)	1 (1–7)	1 (1–35)
Dose 3	2 (1–7)	2 (1–220)	1 (1–7)	1 (1–67)
Systemic events				
Fever				
Dose 1	3 (1–7)	1 (1–32)	4 (1–7)	1 (1–23)
Dose 2	3 (1–7)	1 (1–6)	4 (1–7)	1 (1–24)
Dose 3	2 (1–7)	1 (1–24)	4 (1–7)	1 (1–8)
Fatigue				
Dose 1	2 (1–7)	2 (1–42)	2 (1–7)	2 (1–51)
Dose 2	2 (1–7)	2 (1–203)	2 (1–7)	2 (1–216)

Dose 3	2 (1–7)	2 (1–167)	2 (1–7)	2 (1–192)
Headache				
Dose 1	3 (1–7)	1 (1–44)	3 (1–7)	1 (1–49)
Dose 2	3 (1–7)	1 (1–177)	3 (1–7)	2 (1–86)
Dose 3	2 (1–7)	2 (1–63)	3 (1–7)	2 (1–763)
Vomiting				
Dose 1	4 (1–7)	1 (1–5)	4 (1–7)	1 (1–8)
Dose 2	3 (1–7)	1 (1–32)	4 (1–7)	1 (1–7)
Dose 3	4 (1–7)	1 (1–11)	4 (1–7)	1 (1–7)
Muscle pain				
Dose 1	3 (1–7)	1 (1–50)	3 (1–7)	1 (1–52)
Dose 2	3 (1–7)	1 (1–144)	3 (1–7)	2 (1–126)
Dose 3	3 (1–7)	1 (1–203)	3 (1–7)	2 (1–69)
Joint pain				
Dose 1	3 (1–7)	1 (1–50)	3 (1–7)	1 (1–863)
Dose 2	3 (1–7)	1 (1–162)	3 (1–7)	1 (1–214)
Dose 3	3 (1–7)	1 (1–177)	3 (1–7)	2 (1–40)
Any				
Dose 1	2 (1–7)	2 (1–50)	2 (1–7)	2 (1–863)
Dose 2	2 (1–7)	2 (1–203)	2 (1–7)	1 (1–216)
Dose 3	2 (1–7)	2 (1–203)	2 (1–7)	2 (1–763)

Table S4. Related serious adverse events up to 6 months after dose 3; safety population

	PF-06425090 vaccine (N=8722)[a]		Placebo (N=8718)[a]	
	n[b] (%)	Events,[c] n	n[b] (%)	Events,[c] n
Any	6 (<0.1)[d]	6[d]	1 (<0.1)	3
Gastrointestinal disorders	1 (<0.1)	1	0	0
Pancreatitis	1 (<0.1)	1	0	0
General disorders and administration site conditions	1 (<0.1)	1	1 (<0.1)	3
Death	1 (<0.1)	1	0	0
Injection site hematoma	0	0	1 (<0.1)	1
Injection site pain	0	0	1 (<0.1)	1
Injection site swelling	0	0	1 (<0.1)	1
Musculoskeletal and connective tissue disorders	1 (<0.1)	1	0	0
Polymyalgia rheumatica	1 (<0.1)	1	0	0
Nervous system disorders	1 (<0.1)	1	0	0
Headache	1 (<0.1)	1	0	0
Respiratory, thoracic, and mediastinal disorders	2 (<0.1)	2	0	0
Acute respiratory distress syndrome	1 (<0.1)	1	0	0
Hypersensitivity pneumonitis	1 (<0.1)	1	0	0

[a]N was the number of participants in the safety population who received each study intervention; N differed from the number of participants randomized to each study group (**Figure 1**) because 43 (PF-06425090) and 52 (placebo) participants withdrew before receiving any study intervention, and 2 (PF-06425090) and 1 (placebo) participants were administered a different study intervention regimen after randomization than what had been initially assigned.

[b]Number of participants reporting ≥1 of the specified events.

[c]Total number of events. Each participant may have reported multiple events. For death, n was the total number of AEs reported by participants who died.

[d]An additional related serious adverse event at the end of the study was reported in a PF-06425090 vaccine recipient (psychiatric disorders system organ class and major depression preferred term).
